# Application of Emerging Non-Thermal Processing Technologies: Impact on Characteristics, Efficacy, and Safety of Foods

**DOI:** 10.3390/foods12214040

**Published:** 2023-11-06

**Authors:** Artur X. Roig-Sagués, Antonio J. Trujillo-Mesa

**Affiliations:** Centre d’Innovació, Recerca i Transferència en Tecnologia dels Aliments (CIRTTA), TECNIO CERTA-UAB, Departament de Ciència Animal i dels Aliments, Universitat Autònoma de Barcelona, Travesera dels Turons S/N, Bellaterra, 08193 Barcelona, Spain; toni.trujillo@uab.cat

The development of thermal treatments based on the precepts proposed by Nicolas Appert at the beginning of the 19th century is one of the main milestones achieved to prolong the conservation of food and guarantee its supply to the population even if they are at long distances from production sites [1]. In turn, the application of these treatments has had a fundamental impact on public health, as became evident in the 1930s, when pasteurisation was adopted for the treatment of milk, which saw a drastic drop in the incidence of disease caused by infectious agents present in raw milk [2]. Nowadays, heat-based treatments are the most common way of preserving foods. However, they have some drawbacks, such as undesirable biochemical and nutritional changes in processed products, such as colour changes, a decrease in flavour and aroma, and loss of vitamins [3].

Consumers are increasingly looking for more natural, less processed products, but this is sometimes incompatible with an adequate level of safety. The food industry is constantly evolving, driven by the increasing demand for safe and nutritious food products. The development of emerging technologies in food processing aims to address this specific consumer need for safe, healthy, and minimally processed foods. They have shown promising results in preserving food quality and extending shelf life. These techniques operate at lower temperatures compared to conventional heat treatments, minimising the degradation of sensitive nutrients and bioactive compounds. Furthermore, they can effectively inactivate microorganisms and pathogens, ensuring the safety of food products while also leading to environmentally friendly and sustainable food manufacturing techniques with low energy requirements and reduced water use.

Some of these technologies are based on principles already explored several years ago. The first patents for the use of ionising radiation to kill bacteria in foods were issued in the early 20th century; however, the use of irradiation is still limited nowadays due to psychological and political factors. In the EU, for instance, food irradiation has been regulated since 1999 by a General Directive (Directive 1999/2/EC), but the community list of EU-approved irradiated foods contains only a single class of items: “dried aromatic herbs, spices, and vegetable seasonings” [4]. Less controversial is the use of high pressures for food preservation, the first experiments of which date back to the end of the 19th century. High hydrostatic pressure (HHP), for instance, can be applied to packaged food, avoiding post-treatment contamination. Recently, the European Food Safety Authority [5] has published a report advising that an in-depth analysis be performed on the effect of HPP treatments on inherent compounds and pathogen behaviours in milk, dairy, and other ready-to-eat foods to set generic HHP minimum requirements to assure the safety of these food products. Pulsed electric fields, ultrasounds, and cold plasma are used to exemplify scalable and flexible food manufacturing techniques. Ultra-high-pressure homogenisation and ultraviolet radiation have also been explored in recent years, showing promising results by inactivating effectively food-borne pathogens and extending shelf life but minimising the degradation of sensitive nutrients and bioactive compounds.

Understanding the impact and potential of such technologies on food systems at the cellular level enables the design of tailor-made foods and the establishment of process–structure–function relationships. Based on this knowledge, a completely new process design and the incorporation of these technologies in traditional processes, as well as the generation of improved equipment design, could be possible to maintain or even improve products and fulfil consumer preference, acceptance, and needs [6].

This Special Issue seeks to explore the latest advancements in non-thermal emerging food processing technologies and their impact on various aspects of food processing and product development, with the aim of providing valuable insights into the underlying mechanisms, optimisation strategies, and future prospects for implementing these technologies in the food industry.
